# Role of Stress on Driving the Intestinal Paracellular Permeability

**DOI:** 10.3390/cimb45110581

**Published:** 2023-11-18

**Authors:** Daniel Efrain Molotla-Torres, Fabiola Guzmán-Mejía, Marycarmen Godínez-Victoria, Maria Elisa Drago-Serrano

**Affiliations:** 1 Doctorado en Ciencias Biológicas y de la Salud, Universidad Autónoma Metropolitana, Calzada del Hueso No. 1100, Ciudad de México CP 04960, Mexico; efrainmolotla@gmail.com; 2 Departamento de Sistemas Biológicos, Universidad Autónoma Metropolitana Unidad Xochimilco, Calzada del Hueso No. 1100, Ciudad de México CP 04960, Mexico; 3 Sección de Estudios de Posgrado e Investigación, Escuela Superior de Medicina, Instituto Politécnico Nacional, Plan de San Luis y Díaz Mirón s/n, Ciudad de México CP 11340, Mexico; mgodinezv@ipn.mx

**Keywords:** gut epithelium, paracellular permeability, claudins, occludin, stress, corticosterone

## Abstract

The gut epithelium is a polarized monolayer that exhibits apical and basolateral membrane surfaces. Monolayer cell components are joined side by side via protein complexes known as tight junction proteins (TJPs), expressed at the most apical extreme of the basolateral membrane. The gut epithelium is a physical barrier that determinates intestinal permeability, referred to as the measurement of the transit of molecules from the intestinal lumen to the bloodstream or, conversely, from the blood to the gut lumen. TJPs play a role in the control of intestinal permeability that can be disrupted by stress through signal pathways triggered by the ligation of receptors with stress hormones like glucocorticoids. Preclinical studies conducted under in vitro and/or in vivo conditions have addressed underlying mechanisms that account for the impact of stress on gut permeability. These mechanisms may provide insights for novel therapeutic interventions in diseases in which stress is a risk factor, like irritable bowel syndrome. The focus of this study was to review, in an integrative context, the neuroendocrine effects of stress, with special emphasis on TJPs along with intestinal permeability.

## 1. Introduction

The gut barrier denotes the complex interplay among the biochemical, epithelial, and subepithelial components of the innate and adaptive mucosal immunity that contributes to the intestinal homeostasis [1]. The hallmark function of the gut barrier entails the selective transport of luminal substances toward blood circulation, and it is determined by the epithelium [2]. The epithelium is a physical barrier that acts as a selective filter to allow the entry of nutrients and innocuous molecules and to hamper the access of toxins and harmful substances into the inner milieu [2]. The gut epithelium is a polarized, single-cell layer consisting of enterocytes, Paneth cells, enteroendocrine cells, and goblet cells, and it comprises apical (luminal) and basolateral membrane surfaces. The single epithelial layer drives the gut permeability across transepithelial and/or paracellular pathways [2]. Cellular monolayer components are contiguously joined together by transmembrane glycoprotein complexes known as tight junction proteins (TJPs) that are distributed differentially in each region of the gastrointestinal tract [3]. TJPs are expressed at the most apical extreme of the basolateral membrane and have a role in the regulation of intestinal permeability [4]. Intestinal permeability is a quantitative term that refers to the measurement of soluble molecules across the epithelium from the luminal to vascular side or vascular to luminal side [5]. 

Intestinal permeability is under the control of the engagement of receptors with stress hormones as ligands such as corticotropin-releasing hormone (CRH), glucocorticoids, and catecholamines [6,7,8]. The response to stress encompasses neuroendocrine pathways of the brain–gut axis (BGA), which, in turn, includes the hypothalamic–pituitary–adrenal (HPA) axis and the three major branches of the autonomous nervous system (ANS): the sympathetic nervous system, the parasympathetic nervous system, and the enteric nervous system (ENS) [9]. The HPA axis causes the release of stress hormones via endocrine glands such as corticotropin-releasing hormone (CRH) derived from the hypothalamus, adrenocorticotropic hormone (ACTH) released by the hypophysis, and corticosteroid and catecholamine hormones released by the adrenal glands.

Disarrangements on the TJP location and/or expression that result in alterations in intestinal permeability have been found in inflammatory diseases, with stress being a risk factor that aggravates inflammatory conditions, as seen in irritable bowel syndrome (IBS) and inflammatory bowel disease (IBD) [2,7,10,11,12]. Interplay among BGA, TJPs, and intestinal permeability can be aggravated or triggered by stress, as found in neuropsychiatric disorders like anxiety, depression, impaired social interaction, and cognitive dysfunction, and even in neurodegenerative disease like Parkinson´s disease [13,14]. The impact of stress has been addressed in terms of permeability [15,16,17,18,19] or TJP expression [20,21,22]; hence, this study aimed, for first time, to review the neuroendocrine effects of stress, with a special emphasis on TJPs along with intestinal permeability. This approach, based in preclinical settings, may provide foundations with practical applications for the design of products for balancing intestinal permeability under stress conditions. 

## 2. Tight Junction Proteins: Overview Structure and Function

Cells of the intestinal epithelium are contiguously linked by protein complexes, consisting of tight junctions (TJs), adherent junctions (AJs), desmosomes, and gap junctions. These junctional complexes also confer structural stability to the epithelium, providing a highly polarized and selectively permeable barrier to water, solutes, and other types of larger molecules [4,23]. 

Tight junction proteins are localized in the upper apical region of the basolateral membrane, forming a protein complex embedded in the plasma membrane that interacts with adjacent cells [24]. Within the transmembrane proteins that make up the TJs are occludin; claudins; tricellulins; myelin and lymphocyte protein (MAL); relevant proteins for vesicle trafficking, for example, membrane link domain 3 (MARVEL D3); and members of junctional adhesion molecules (JAMs). The latter are anchored to the cytoskeleton of epithelial cells via scaffolding proteins as zonula occludens (ZO), ZO-1, ZO-2, and ZO-3; cingulin; and simplequine [24]. Occludin regulates the assembly of TJPs [25] and acts as an accessory protein for claudins [26]. Tricellulins are located in bicellular TJs, but predominantly in tricellular TJs [27], where they form a central pore that allows the passage of solutes (≤10 kDa) [24].

Claudins are a family of proteins that are widely expressed in many tissues, for example, the skin barrier, proximal renal tubule, blood–brain barrier, and intestinal barrier [28]. Regarding intestinal epithelial tissue, claudins are the main structural components of TJ strands [26]. Claudins have two extracellular loops (ECL 1 and ECL 2) that form strands in the TJs [29,30]. The charge of amino acids that make up ECL 1 and ECL 2 is responsible for the barrier properties or formation of pore of the claudins; moreover, the permeability of the TJ depends largely on the claudin distribution pattern and the interactions between the claudins. These encompass the following: cis (ELC of the same cell), trans (ELC of adjacent cells), homophilic (ELC from the same claudin family), and heterophilic (ELC from different claudin families) interactions [31,32]. Currently, 27 types of claudins are known, and functionally, they can be classified into (i) pore-forming claudins that contribute to increasing intestinal permeability, such as claudin-2 (cldn-2), -10a, -10b, -15, and -17 [30]; (ii) barrier-forming claudins (or pore-sealer claudins) that decrease the intestinal permeability, such as cldn-1, -3, -4, -5, -8, -11, -14 and -19 [32,33]; and (iii) claudins with ambiguous functions that have a double effect by increasing or decreasing intestinal permeability, such as cldn-4, -7, -8 and -16 [32]. Some claudins, such as cldn-6, -9, -12, -13, -18, and -20 to -27, have been little studied; therefore, there is little information about their functions [30].

Claudins are expressed throughout the intestine [3]; however, they have a regionalized distribution in each segment of the intestinal tract. In mice, some claudins show a divergent gradient of expression; for example, cldn-8 location increases while cldn-15 decreases from the small intestine to the colon. At the ileocecal region, cldn-2, -5, -7, and -10 are highly expressed, while cldn-2, -3, -7, and -15 are evenly distributed along the whole length of the intestinal tract [34]. In rats, the barrier-forming claudins (cldn-1,-3,-4, -5, and -8) are expressed to a major extent in the colon compared with the jejunum and ileum, while pore-forming claudin expression (cldn-2 and 7) is greater in the jejunum and ileum than the colon [33]. In the human colon, cldn-1, -2, -3, -4, -5, -7, and -8 are expressed [33]. In humans, cldn-1, -2,-4,-5, and -12 have been used as diagnostic markers for collagenous colitis, ulcerative colitis, Crohn´s disease, and pouchitis [33].

Adherent junction proteins complexes (AJPs) are present below the TJs, toward the basolateral side. They are made up of molecules belonging to the cadherin (Ca^2+^-dependent) and nectin (Ca^2+^-independent) families [35]. Alike TJPs, AJ proteins are anchored to the cytoskeleton through the perijunctional F-actin ring [36]. The main function of AJPs is to maintain cell–cell adhesion. Adherent junction protein complexes also function as a hub for cell signaling and the regulation of gene transcription [37].

Desmosomes are made up of desmoglein and desmocollin families of desmosomal cadherins, and connecting proteins such as desmoplekin and keratin [24], located below the AJs toward the basolateral side. Like AJPs, desmosomes form similar cell–cell adhesions, both dependent and independent of extracellular Ca^2+^. Calcium-dependent interactions encompass homodimeric desmocollin:desmocollin and heterodimeric desmoglein:desmocolin. The latter transits to being calcium-independent as desmoglein2:desmocollin2, which confers stronger intercellular adhesion [35].

Gap junctions are made of hexamer assemblies of integral tetraspan membrane proteins [38], located below the desmosomes toward the basolateral side. Gap junctions form intercellular channels that allow the paracellular transfer of ions and small molecules [24].

## 3. Methods of Intestinal Permeability Evaluation

**Figure 1:**
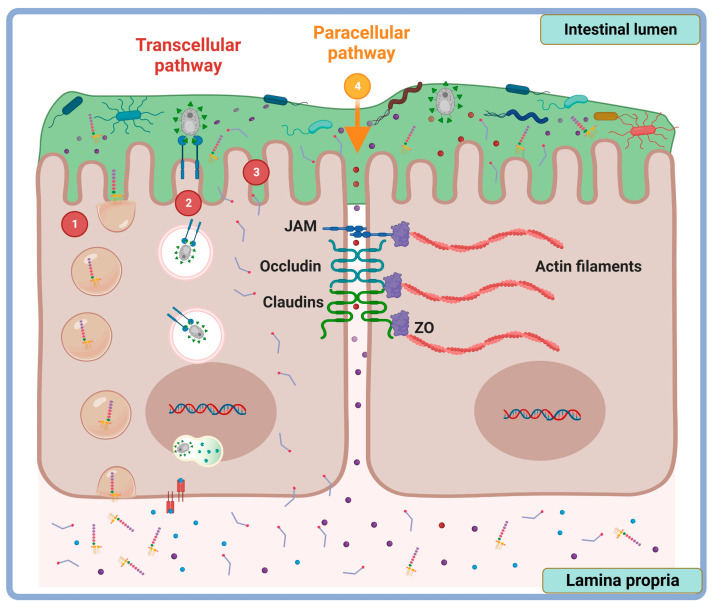
Intestinal transcellular and paracellular permeability. Transport mechanisms of the epithelial cell monolayer involve the passage of particles through (1) endocytosis and (2) phagocytosis or solutes via (3) facilitated transport. The paracellular route allows the passage of ions and small molecules between the spaces of the lateral membrane of the epithelial cells (4). JAM, junctional adhesion protein; ZO, zonula occludens. Created at Biorender.com.

Intestinal permeability can be categorized according to the route through which substances cross the epithelial barrier. There are two pathways by which intestinal permeability occurs: the paracellular route, which refers to the passage of molecules through the intercellular spaces; and the transcellular route, which denotes the passage of agents across the epithelial cells. The permeability through each pathway is conditioned by the nature of the “diffuser” (size, charge, and/or polarity) [24] (Figure 1). 

The transcellular route represents the main pathway by which small fat-soluble compounds (passive diffusion) [39], water-soluble compounds (facilitated transport by permeases) [40], particles (endocytosis and phagocytosis), and soluble macromolecules (pinocytosis) are transported [41].

The paracellular route refers to the transport of solutes or molecules through the intercellular spaces of the epithelium. The paracellular pathway takes place for small- and medium-sized hydrophilic molecules, less than 600 Da; for this reason, protein-sized molecules, and macromolecules such as antigens, are impermeable to this barrier [42]. This route is of special interest in terms of intestinal permeability, since it contributes significantly to the transport of water and solutes through the intestinal epithelium [43].

The paracellular and transcellular intestinal permeability can be measured by non-invasive methods in humans [44]. In the next section, several preclinical assays are described. 

### 3.1. Semiquantitative Assessment of Intestinal Barrier Integrity

Several methods are available for the microscopic assessment of TJP in the intestine [45]. Histological analysis of the intestinal barrier by light microscopy using hematoxylin and eosin (H&E) staining allow us to evaluate tissue integrity, the presence of mucosal ulcerations, and inflammatory infiltrate that could contribute to an increase in permeability [45]. Hematoxylin and eosin staining is a standard method applied for the histological observation of intestinal biopsies from patients with intestinal inflammatory diseases [46]. Similar to H&E staining, which is very useful for evaluating the gross morphology, electronic microscopy is a powerful method for the detailed observation of the architecture of junctional complexes in epithelial cells, with clinical implications for diagnosis and therapy. Electronic microscopy enables a detailed analysis of intestinal barrier damage, as reported in irritable-bowel-syndrome-like disorders [46]. Furthermore, the immunohistochemical (IHQ) method is the best choice to visualize the extent of the in situ location and distribution of TJPs, and the structural integrity of the intestinal epithelium. Immunohistochemical analysis requires the use of a specific fluorescent-dye-conjugated antibody to detect TJPs of interest, as documented in duodenal samples from patients with obstructive jaundice [47]. Aside from histological methods, TJPs can be estimated by assessing the protein expression using the Western blot technique, as documented in a human trial of patients with intestinal barrier abnormalities [48], or by evaluating the mRNA levels of TJPs by RT-qPCR, as has been assayed in colonic tissues from healthy human volunteers [49].

Currently, many tests have been implemented to measure paracellular and/or transcellular intestinal permeability in vivo and in vitro. The techniques are designed for the quantification of solutes that cross the epithelial membrane, either from the blood to the intestinal lumen, or from the lumen to the blood [45].

### 3.2. Assessment of Intestinal Permeability In Vivo

#### 3.2.1. From the Intestinal Lumen toward the Blood

Permeability assessment from the intestinal lumen toward the blood consists of the administration of non-metabolizable markers via enteral and jejunal loops. The transport of these markers across the epithelial barrier is increased in pathological conditions when the intestinal permeability is altered [50]. Therefore, intestinal permeability relies on the quantification of markers delivered by the enteral route in the blood, specific organs, or urine samples. There are several types of markers, for example, (i) sugars, (ii) fluorochromes, (iii) dyes (Evans blue), and (iv) polyethylene glycol (Table 1). The choice of these markers depends on the type of permeability assessed, i.e., transcellular or paracellular permeability. The evaluation of intestinal permeability with sugars is widely used for clinical diagnosis. One limitation of this approach depends on factors, including gastrointestinal motility, that may lead to the inaccuracy of permeability measurements [51].

#### 3.2.2. From the Blood to the Intestinal Lumen

In these techniques, the markers are administered via the intravenous (I.V.) route to enter into the blood circulation and then pass through semi-permeable membranes to the intestinal lumen; markers can be measured in the intestinal tissue or stools. Although the intestinal permeability for these markers is moderate, under pathological conditions, it is substantially increased. The markers that are widely used for the evaluation of intestinal permeability from the blood to the lumen are (i) albumin, (ii) Evans blue, and (iii) ^51^Cr–EDTA (Table 2). Compared to the techniques used to evaluate the permeability from the lumen to the blood, these assays to evaluate the permeability from the blood to the intestinal lumen are not influenced by gastrointestinal motility, providing reliable results.

### 3.3. Evaluation of Intestinal Permeability In Vitro

In vitro models based on cell cultures that mimic the structure, physiology, and function of the epithelial tissue have the advantage of creating specific, controlled, and repeatable conditions [61]. Cell lines are derived from human colon carcinomas, such as the cancer coli-2 (Caco-2) cell line [62]. These cell lines have several morphological characteristics similar to small intestine enterocytes, such as a polarized cylindrical shape, microvilli on the apical region, and the intercellular junctions formed by TJPs [63]. In the case of T84 cells, their treatment with transforming growth factor (TGF)-ꞵ allows their differentiation in induced crypt-like cells [64]. Moreover, the T84 cell line is superior to the Caco-2 cell line as a model of colonocytes for in vitro assays to evaluate the effects of compounds on the integrity of the intestinal barrier, as well as the absorption, metabolism, and bioavailability of drugs through the epithelial barrier [65]. In addition, HT29 treatment with methotrexate (MTX) generates the differentiated mucus-producing cells, which allows for evaluation of the effect of food compounds on the mucus and/or the assays of adhesion of the microbiota [62]. In addition to the cancer-derived cell lines, primary cell lines isolated from healthy tissues are usually used for in vitro assays; these primary cells are isolated by the limiting dilution technique that allows better preservation of the original anatomical features [66]. Some functional cell lines, for example, epithelial (H4) cells derived from healthy human fetal small intestine, are used in assays for determining the moderate and high transepithelial electrical resistance (TEER) and transepithelial electrical potential (TEEP) [66,67]. Intestinal porcine enterocyte cell line J2-3 (IPEC-J2-3) and IPEC-J2-9, derived from the neonatal pig small intestine, are used to evaluate high TEER and neonatal gut development. Non-carcinogenic porcine-derived enterocytes (CLAB) derived from pig small intestine enterocytes are used in paracellular transport assays [66].

### 3.4. Assessment of Intestinal Barrier Function Ex Vivo

Ex vivo models are living tissues that are maintained in an artificial environment. Ex vivo evaluation of the epithelial barrier function is a simplified and controlled way to understand the behavior and mechanisms involved in the function of the epithelial barrier. These assays represent an alternative approach to the use of animals, circumventing ethical problems and reducing the economic cost [61]. Unlike the in vitro models based on monocellular cultures, the ex vivo models reflect the interaction of different types of epithelial cells, as well as their densities according to the intestinal region [68]. Evidently, the main limitation of ex vivo models is that they do not reflect the physiology of the diseases studied in animal models [69].

#### Ussing Chamber Technique

The Ussing chamber assay can measure the paracellular permeability of different types of epithelial tissues from cell cultures and ex vivo tissues [54]. The TEER measures the resistance that the tissue exerts against the flow of cations and/or anions what reflects the functional and structural integrity of the TJPs. Resistance is measured by applying a constant electric current through the membrane, which generates a potential difference; the latter can be interpreted using Ohm’s law and is expressed as Ω × cm^2^. The Ussing chamber assay is also used to evaluate the transport of solutes such as: EDTA, mannitol, sucrose, inulin, and polyethylene glycols or dextran molecules of variable sizes [54]. The main advantage of the Ussing chamber technique relies on the possibility to study the absorption and immune responses of the different regions (duodenum, jejunum, ileum, or colon) of the gastrointestinal tract [68]. The Ussing chamber has disadvantages including the limited viability and availability of tissue samples for analysis and high individual variability that provide inconclusive evidence. The Ussing chamber assay is a hard-working and time-consuming method that requires high technical skills; moreover, its application is mostly limited to running experiments on a laboratory scale for analyzing a small number of samples [68]. The Ussing chamber technique provides critical information concerning the functional and structural integrity of the intestinal barrier as well as for presumable pathophysiologic and neuroendocrine mechanisms, where several factors disrupt the transcellular and paracellular permeability. The Ussing chamber assay has been used to evaluate intestinal permeability in in vitro Caco2 culture assays [70] and in colonic samples from healthy individuals [49,71]. Also, the Ussing chamber assay has clinical implications in the diagnosis of IBS [72,73,74,75].

## 4. Stress Modulation Pathways on Permeability and TJP

All organisms maintain a complex and dynamic balance that is constantly exposed to external and internal stimuli, called stressors. To re-establish homeostasis, the organism generates a series of physiological responses that involve the production and release of various neuroendocrine mediators [76]. Stress is a response that the organism triggers when it detects a stimulus that threatens its homeostasis. The stimulus can be physical or psychological and of varying duration; consequently, the evoked physiological response is not always the same or of the same intensity [76]. Depending on the duration and the type of stimulus, the effects of stress can be harmful to the health of the individual. Acute stress occurs for short periods ranging from minutes to hours, while chronic stress persists for days, weeks, and even months. On the other hand, stressors can be classified according to their intensity, which is measured by the levels of hormones or neurotransmitters that are released after the stimulus [76].

**Figure 2:**
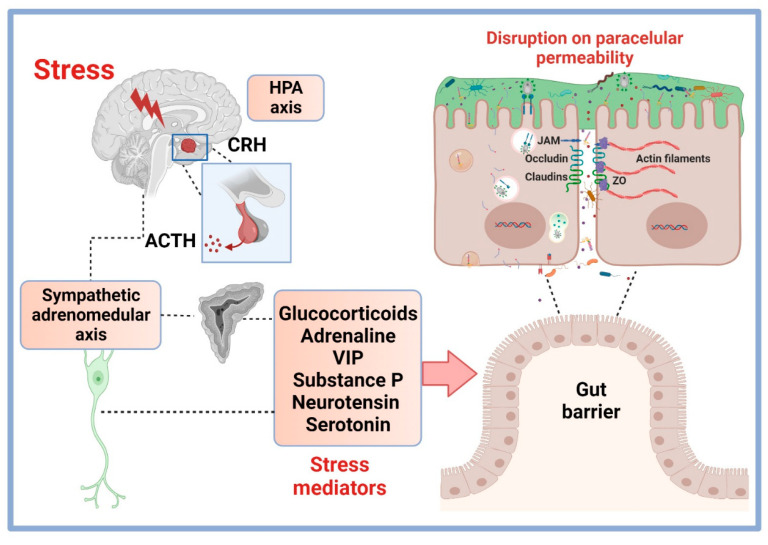
Stress mediators and their effects in intestinal paracellular permeability. Stress response activates both hypothalamic–pituitary–adrenal (HPA) and sympathetic adrenomedullary axis to promote the secretion of hormones and neurotransmitters that induce paracellular permeability disruption. CRH, corticotropin-releasing hormone; ACTH, adrenocorticotropic hormone; VIP, vasoactive intestinal peptide; JAM, junctional adhesion molecule; ZO, zonule occludens. Created at Biorender.com.

The physiological response to stress involves efficient communication between different systems that aim to maintain physiological integrity, even in the most demanding circumstances. Among these systems, we can mention the ANS (including the sympathetic nervous system, the parasympathetic nervous system, and the ENS) and the HPA axis [77]. The ANS provides the most immediate response upon exposure to the stressor through the sympathetic and parasympathetic pathways [78]. A stressor induces rapid physiological alterations, for example, increases in blood flow, heart rate, and the excitability of the cardiovascular system. The brain stem receives signals from homeostatic disturbances such as decreased blood flow, visceral pain, and inflammation. The sympathetic response to these signals of damage involves the actions of the reflex arc and regions in the spinal cord (ventrolateral rostral medulla) communicate with sympathetic preganglionic neurons (Figure 2). The parasympathetic response to stress occurs via the nucleus ambiguus and the dorsal motor nucleus of the vagus nerve, positioned in the nucleus tractus solitarius. The ENS response involves the release of neuropeptides and neurotransmitters that exert their effects through the activation of receptors located in the intestine. Activation of these pathways results in the rapid secretion of adrenaline, norepinephrine, acetylcholine, and other neurotransmitters in the central and peripherical systems. Activation of the HPA axis begins in the paraventricular nucleus of the hypothalamus, which induces the production of corticotropin-releasing hormone (CRH). This hormone is transferred by the portal system to the pituitary gland, where it stimulates the synthesis of pro-opiomelanocortin. This is subsequently processed and converted into adrenocorticotropin hormone (ACTH) to stimulate the synthesis of glucocorticoids in the adrenal cortex [76]. In addition to the classic hormones and neurotransmitters (adrenaline and glucocorticoids), stress induces the release of other mediators such as neuropeptides (substance P (SP) and neurotensin), cytokines, growth factors, and neurotransmitters (acetylcholine, serotonin, and dopamine), that are produced by nerve terminals or by endocrine glands. These mediators regulate the homeostasis in the organism and their effects on increasing paracellular permeability has been demonstrated in various studies [15]. The above mechanism is caused by the rearrangement of actin fibers and the consequent internalization/recycling of TJPs. Figure 2 depicts the sympathetic and HPA axes as major pathways of stress activation.

### 4.1. HPA Axis Stress Hormones and Permeability 

Glucocorticoid (GC) hormones are the end-point targets of the HPA axis activation, one of most paradigmatic endocrine routes of stress regulation addressed in terms of permeability and TJPs [6,8]. Controversial stress-induced glucocorticoid up- and down-modulatory outcomes on intestinal permeability depend, in part, on the conditions of study (in vitro versus in vivo), animal stress model, stress frequency/intensity, among other factors, but also highlight the multilevel points of regulation. In animal models, it has been found that stress-induced GC elicitation breaks down the intestinal permeability and/or TJP remodeling. Regionalized analysis of the stress-induced corticosteroid response on permeability and TJP expression in the small intestine and colon was documented in rats who underwent (i) subacute stress/24 h by small cage housing with limited movement, (ii) chronic stress for 1 h/14 days by crowding, and (iii) the combination of subacute and chronic stress. TEER was decreased by the three stress models in the jejunum or only by the combination of subacute and chronic stress in the colon. In the jejunum, increased mRNA levels of cldn-1, -5, -8, occludin, and ZO-1 by subacute stress, or cldn-2 by combined stress models, correlated negatively with TEER. All three stress modalities increased the circulant corticosterone and none of them affected ACTH response. Corticosterone elicitation correlated negatively with the jejunal TEER decrease and positively with the jejunal cldn-5 mRNA increase. Changes in TJP expression or their correlation with the TEER decrease or corticosterone elicitation were not observed in the colon, although mast cell infiltration increased by chronic stress and combined subacute/chronic stress only in the colon, not in the jejunum [79]. Data indicated that the extent and frequency of the stress-induced glucocorticoid response divergently modulates intestinal permeability and TJP expression in the small intestine versus the colon. This might result from either up- or down-immunomodulatory actions of glucocorticoids synthetized locally by steroidogenic enzymes [80] and/or the extent of glucocorticoid receptor location, which has been found to be greater in the colon than the jejunum [81]. 

The outcome of the stress-induced glucocorticoid response in terms of regionalization was also documented in rats who underwent chronic water avoidance stress (1 h/10 days) [81]. In this contribution, the effects of stress on the increase in corticosterone as well as on the decrease in cldn-1, occludin, and ZO-1 protein were observed in the colon, but not in the jejunum. These effects were reversed by RU-486 treatment (a glucocorticoid receptor antagonist) in stressed rats or in rats injected with corticosterone and treated with RU-486 [81]. Furthermore, the impact of chronic water avoidance stress or corticosterone treatment on the down-modulation of corticosterone receptor protein (currently denoted as NR3C1) was prevented by RU-486 treatment in the colon, but not in the jejunum. Additionally, chronic water avoidance stress effect on the increased permeability of polyethylene glycol-400 (PEG-400 molecular mass) was prevented by RU-486 in the colon, but not in the jejunum. Chronic stress did not alter the permeability of PEG-4000 and PEG-35,000 in both segments; however, permeability was lower in the colon than the jejunum for PEG-4000, while both segments showed no differences in permeability for PEG-35,000. The data indicated that GC stress response via the glucocorticoid receptor (GCR) controls colonic permeability to molecules with a molecular mass less than 4000 kDa and controls visceral hyperalgesia, providing a rationale for novel treatments that target colonic TJPs [81].

A regionalized analysis of apical-crypt levels of cldn-1 (a barrier-enhancer claudin) expression in the colonic crypts of rats who underwent chronic water avoidance stress (1 h/10 days) demonstrated a delicate molecular mechanism, upon which, chronic stress regulates the intestinal barrier function. In this assay, the glucocorticoid receptor NR3C1 and the basic helix–loop–helix (bHLH) transcription repressor factor hairy and enhancer of split 1 (HES1) were analyzed [82]. NR3C1 is expressed prominently in the colon and its expression is abrogated by a stress-induced corticosterone increase or by corticosterone treatment. NR3C1 is located at the apical level of fully differentiated epithelial villi, and it controls cldn-1 transcription via binding to glucocorticoid response elements (GREs) on the CLDN1 promoter at its transcription start site (TSS). Regarding HES1, it is a nuclear protein factor required for the differentiation of neurons and epithelium enterocytes. It is located prominently at the base of colon crypts (undifferentiated cells) and binds the N-box elements on the CLDN1 promoter. In this study, chronic stress down-modulated cldn-1, NR3C1, and HES1 mRNA/or protein levels and prevented the binding of NR3C1, but not HES1, to CLDN1 promoter in rat colonic crypts. Furthermore, RU-486 treatment reversed this effect. In vitro assays carried out in Caco-2 cell cultures with either a low cell density or those treated with cortisol caused the decrease in TEER values as well as HES1 expression. [82]. Overall data analysis suggested that chronic stress down-modulated HES1 that was high in the colon crypt, proliferating enterocytes and down-modulating cldn-1 and NR3C1, which were high in colon luminal enterocytes. The proposed mechanism relies on glucocorticoid-activated NR3C1 inhibiting (“switches off”) HES1 promoter transcription and activating (“switches on”) CLDN1 promoter transcription via GRE. Thus, stress-induced glucocorticoid elicitation impairs colon epithelial function by interfering with the CLDN1–HES1 intrachromosomal communication via NR3C1/GRE modulation [82].

In vitro assays have provided pivotal insights regarding the underlying mechanisms upon which interaction of GC with its GCR activates signal pathways that control intestinal permeability [83]. It is important to underline that, in some cases, in vivo versus in vitro assays provide contradictory mechanisms. In in vitro Caco-2 culture assays, glucocorticoids (prednisone and/or dexamethasone) did not alter TJ intestinal permeability measured as TEER or paracellular permeability measured with inulin (5 kDa molecular mass), but they did counteract the effect of tumor necrosis factor α (TNFα) in terms of both the TEER decrease and paracellular permeability increase. Molecular analysis indicated that prednisolone elicited the activation of the cytoplasmic GC–GCR complex, its nuclear translocation, and its binding to the glucocorticoid response element (GRE) DNA site. All of these changes were blocked by the GCR antagonist RU-486. Furthermore, prednisolone counteracted the effect of TNFα on the increase in myosin light chain kinase (MLCK) protein expression, necessary for mediating the TNFα-induced increase in TJ intestinal permeability. MLCK is a serin/threonine-specific protein kinase that phosphorylates the myosin light chain (MLC); elevated levels of MLCK and phosphorylated MLC (pMLC) are associated with stress-induced intestinal permeability dysfunction [15,17]. Data suggested a presumable mechanism based on the fact that glucocorticoids inhibit the effect of TNFα in terms of a paracellular permeability increase through the GC–GCR complex activation and its binding site on the MLCK promoter region; the latter suppresses the TNFα-induced increase in MLCK gene activity, proteins, and the subsequent opening of the intestinal TJ barrier [83].

The findings of studies using Caco-2 cell cultures support the interpretation regarding glucocorticoids in terms of regulating claudin expression and intestinal permeability via mitogen-activated protein kinase (MAPK) phosphatase 1 (MKP1) [84]. MKP1 causes the dephosphorylation and inactivation of MAKP and the down-regulation of p38 and JNK; MKP1 mediates the anti-inflammatory effect of glucocorticoids [85]. In this Caco-2 cell setting, dexamethasone increases TEER in a GR-dependent manner that was suppressed by RU-486 treatment; dexamethasone did not cause changes in paracellular permeability measured by fluorescent tracer lucifer yellow (450 kDa, 9.9 Ǻ) or fluorescence-labeled dextran (4 kDa) [84]. Dexamethasone decreased cldn-2 (pore-forming claudin) and increased cldn-4 expression (pore-sealing claudin) but caused no changes in occludin, ZO-1, cldn-1, cldn-7, and cldn-8. In contrast, cldn-5 was undetectable in fully differentiated Caco-2 cells. The decrease in cldn-2 induced by dexamethasone down-modulated the paracellular permeability of sodium chloride, and this effect was antagonized by the administration of RU-486. In addition, dexamethasone increased the enzymatic MKP1 activity resulting in MAKP inactivation; treatment with the MKP1 inhibitors triptolide and sanguinarine antagonized the effect of dexamethasone on the increase in TEER, cldn-2, and cldn-4 in a dose-dependent manner. However, pharmacological inhibition of p38 or MEK1/2, using BIRB796, U0126, or SB203508 to block the downstream MAPK signal, did not increase TEER. Interleukin-1β (IL-1β) combined with TNFα decreased TEER and increased cldn-2 without affecting cldn-4. This effect on cldn-2 expression was antagonized with dexamethasone. Mechanistically, the data indicated that GC antagonized the cytokine-weakening effects on TEER and TJ permeability by forming a GC–GCR complex to be targeted by glucocorticoid response element (GRE) DNA and block MAPK-induced TNFα-promoter expression. As is already known, inflammatory cytokine signal pathways result in the expression of MLCK activity involved in TJ opening [84].

Luminal colonization of commensal bacteria is a critical factor underlying the apparent discordant findings regarding the stress-induced GC on intestinal barrier function, addressed under in vitro versus in vivo environments. Complex interplay between the stress-induced glucocorticoid response and intestinal bacteria or bacterial-derived products on intestinal permeability and TJP expression has been evidenced in stress assays in mice [86,87]. 

**Figure 3:**
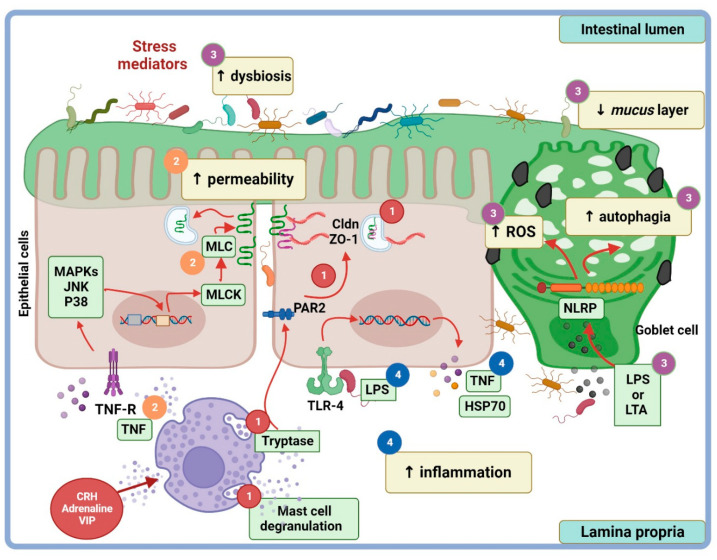
Cellular and molecular mechanisms of stress on intestinal paracellular permeability. (1) Corticotropin-releasing hormone (CRH) and other stress mediators induce mast cell degranulation, generating the release of tumor necrosis factor α (TNFα) and tryptase that promote proteinase-activated-receptor-2 (PAR2) degradation. This results in the re-localization of zonula occludens-1 (ZO-1) within the epithelial cell, increasing the intestinal paracellular permeability. (2) TNFα promotes the increase in myosin light chain kinase (MLCK) expression and, subsequently, the phosphorylation of myosin light chain (MLC). This pathway modifies actin filaments and internalizes claudins, increasing intestinal paracellular permeability. (3) Dysbiosis and the disruption of permeability allow the entrance of pathogenic bacteria and their components such as lipopolysaccharide (LPS) and lipoteichoic acid toward the basolateral side of the epithelial monolayer cell. Lipopolysaccharides and lipoteichoic acid interact with receptors, resulting in NOD-like receptor family pyridine-domain-containing (NLRP) inflammasome activation that promote the oxidative stress and autophagia, promoting a decrease in the mucus layer. (4) LPS interaction with the epithelial cell toll-like receptor 4 (TLR-4) results in its activation and the induction of the cytokine production such as TNFα. This facilitates the inflammatory state in the intestinal barrier. ROS, reactive oxygen species; LTA, lipoteichoic acid; JNK, Jannus kinase; VIP, vasoactive intestinal peptide; HSP70, heat shock protein 70; Cldn, claudin; TNFR, TNF receptor. Created at Biorender.com.

In an experimental setting of acute-restraint (2 h) stress in male C57BL/6 mice, the impact of stress-induced heat shock protein 70 (HSP70) expression on intestinal permeability was analyzed [86]. Heat shock proteins have protective roles in the gut epithelial cells and their expression is mediated by commensal bacteria through the ligation of the bacterial lipopolysaccharide (LPS) with the host toll-like receptor 4 (TLR4) [88]. Findings evidenced that acute stress enhanced colonic HSP70 protein production, and this effect was antagonized by RU-486, suggesting the role of the GC–GCR signal pathway. Elimination of commensal bacteria by an antibiotic cocktail abolished the effect of acute stress on the HSP70 protein increase. Furthermore, oral LPS treatment in bacterial commensal-depleted colons from mice treated with an antibiotic cocktail, restored the effect of acute stress on HPS70 protein elicitation. Acute stress did not affect in situ TLR4 protein expression in the colon but increased Alexa-LPS 548 (as a tracer for permeability) toward the lamina propria and decreased ZO-1 protein location in the colon. Findings have indicated that HSP70 elicitation by acute stress depends on endogenous GCs and luminal bacteria and/or their bacteria derivatives. It is known LPS–TLR4 ligation requires disruption of TJP junctions to favor the interaction of LPS released at the luminal surface with TLR4 located on intestinal basolateral membrane [89] (Figure 3, mechanism 4). Hence, data suggested a presumable mechanism, upon which stress-induced GC elicitation down-modulated intestinal permeability. Moreover, ZO-1 expression relies on favoring the passage of LPS to the lamina propria and its ligation with TLR4, resulting in a signal pathway to generate HSP70 [86].

The passage of bacteria-derived LPS from the lumen toward the lamina propria, and eventually, to the blood circulation, drives local and systemic inflammation responses that represent a serious life-threating concern [90]. Therefore, the intestinal epithelium has developed tolerance to LPS, i.e., a reduced capacity to respond to LPS following a first exposition to the stimulus resulting from the ligation of LPS with TLR4. Thus, mice underwent chronic water avoidance stress (1 h/10 days) in an experimental setting and the impact of stress-induced corticotropin-releasing factor (CRH) on disturbing the epithelial LPS tolerance was documented [87]. In this assay, stress up-modulated the endocrine responses of serum corticosterone, ACTH, norepinephrine, and prolactin, in addition to the expression of cldn-2 in the colonic tissue, as well as cldn-2 and TLR4 mRNA/protein. Permeability to horseradish peroxidase (HRP), TEER (Isc μA/cm^2^), and conductance (mS/cm^2^) were found to be increased in the Ussing chambers mounted with the intestinal epithelium of stressed mice and treated with LPS. All of these changes were abrogated by pretreatment with anti-cldn-2 polyclonal antibody (pAb). Furthermore, stress-induced TLR4 expression was abrogated with the α-helical CRH antagonist. The data suggested that a mechanism relies on that chronic-stress-induced CRH response, resulting in the overexpression of TLR4. The ligation of TLR4 with LPS increased the expression of the pore-forming cldn-2, which was associated with gut barrier weakening and, ultimately, the breaching of the established tolerance to LPS. The findings may support increased cldn-2 expression in colonic epithelial cells in diseases in which stress is a risk factor, such as irritable bowel syndrome [87]. The outcome of the LPS–TLR4 signal pathway on gut barrier breaching and visceral sensitivity can be controlled with natural and sustainable products such as a mixture of probiotics. This has been documented in in vitro assays of T84 cell cultures and in C57BL/6 mice who underwent chronic water avoidance stress (1 h/4 days) [91].

The impact of stress and dietary habits on dysbiosis (i.e., disarrangements in the abundance and colonization of commensal bacteria communities) has been associated with intestinal permeability disturbances, as observed with chronic diseases like IBD. In this context, analysis of the microbiota and the stress-induced GC response on LPS translocation, gut permeability, and TJP expression was addressed in female C57BL/6 mice who underwent chronic restraint stress (6 h/14 days) and were fed high dietary fructose [92]. The data indicated that chronic stress down-modulated body weight, anti-inflammatory interleukins, occludin, cldn-1, cldn-3, mucin-secreting goblet cells, antioxidant enzymes (glutathione peroxidase, superoxide dismutase, catalase), and autophagia, while up-regulating parameters of damage like colonic FITC–dextran permeability, serum corticosterone/norepinephrine, serum LPS derived from commensal microbiota, proinflammatory cytokines, and apoptosis (Figure 3, mechanism 3). Most stress-associated effects were exacerbated by a high-fructose diet derived from alteration in the abundance of gut microbiota. Dysbiosis led to an increase in histamine and a decrease in taurine that, in turn, were related to alterations in the components of epithelial and subepithelial barrier components, the destruction of NLRP6, and a reduction in autophagia. The NOD-like receptor family pyridine-domain-containing (NLRP6) inflammasome acts as an intracellular sensor that regulates the abnormal expansion of commensal microbiota communities [93]; autophagia is a pivotal event to maintain the microbiota diversity [94]. Down-modulation of NLRP6 and autophagia lead to the accumulation of reactive oxygen species [94]. Reactive oxygen species induce proinflammatory cytokine generation, driving a breach in the gut barrier, resulting in local and systemic inflammation [95]. Depletion of the microbiota with antibiotics and then re-colonization with *Akkermansia (A.) muciniphila* in mice under conditions of chronic stress restored the expression of NLPR6 inflammasome and autophagia in goblet cells, TJP expression, and intestinal permeability [92]. *A. muciniphila* is a Gram-negative, strictly anaerobic bacterium belonging the phylum *Verrucomicrobia*, which contribute to gut barrier remodeling by degrading the mucus layer [96]. Mechanistically, data have indicated that chronic stress and a high-fructose diet lead to dysbiosis and the generation of microbial metabolites, reactive oxygen species, apoptosis, and so on, involved in intestinal barrier damage; moreover, *A. muciniphila* restores the intestinal barrier under stress conditions by restoring beneficial metabolites at the intestinal milieu [92].

### 4.2. Additional Stress Pathways on Paracellular Permeability

The growing body of compelling evidence supports the outcome of stress on TJP remodeling and the intestinal permeability regulation by key stress pathways. 

(i) Peroxisome-proliferator-activated receptors (PPARs), including PPARα, PPARβ/δ, and PPARγ isotypes, are members of the nuclear hormone receptor superfamily. They act as ligand-inducible transcription factors that play a wide array of roles including in energy consumption and intestinal homeostasis [97,98]. PPARα, PPARβ, and PPARγ are expressed in the intestine and activated fatty acid catabolism [98]. PPARα is involved in mitochondrial and peroxisomal β-oxidation and inhibits inflammation by through the repression of the nuclear factor κB (NFκB) and the activation of protein 1 (AP-1) [97]. PPARγ displays an anti-inflammatory role by inhibiting the MAPK–NFκB signaling pathway [98], and its impact on disturbing stress-induced permeability was analyzed in the colons of rats who underwent acute/chronic restraint stress [99]. Acute (6 h/1 day) and chronic stress (6 h/5 days) triggered myeloperoxidase (MPO) activity, the expression of the enzymes cyclooxygenase (COX2) and nitric oxide synthase 2 (NOS2), and the generation of endogenous prostaglandin E2 (PGE2 and PGJ2), and it increased the intestinal permeability (^51^Cr–EDTA), translocation of luminal commensals to mesenteric lymph nodes (MLN), and tight junction openings. Most effects of acute stress were reversed with prior intraperitoneal injection of 15-deoxy-Δ12,14 prostaglandin J2 (15d-PGJ2), a PPARγ natural agonist, and rosiglitazone, a PPARγ synthetic agonist; however, the effects of both agonists were abrogated by theT0070907 PPARγ antagonist. These findings highlighted the impact of stress on generating an oxidative and inflammatory milieu in the colon, favoring the breaching of gut permeability and bacterial translocation. 

An additional contribution addressed the role of PPARα against the stress alterations on the gut barrier in PPARα-knock-out mice who underwent acute board immobilization stress [100]. The results evidenced that, in the absence of PPARα, stress caused a significant increase in fecal pellet output and intestinal permeability (lactulose/mannitol urine clearance); in the ileum and/or colon, stress increased histological disarrangements, inflammation, TNFα, Fas Ligand (FasL), Tunnel+ cells, caspase 3, and proapoptotic Bax protein, while stress decreased ZO-1, occludin, β-catenin, and the anti-apoptotic Bcl-2 protein. The findings suggested that PPARα engagement with endogenous ligands may provide signals that counteract the stress-induced apoptosis activation to ameliorate intestinal permeability disfunction [100].

(ii) The endocannabinoid system comprises the cannabinoids 2-arachidonoylethanolamine (AEA) and 2-arachidonoylglicerol (2-AG), cannabinoid receptor 1 (CBR1) and CBR2, and enzymes such as fatty acid amide hydrolase (FAAH) and monoacylglycerol lipase (MAGL). While 2-arachidonoylethanolamine interacts with CBR1 and is degraded by FAAH, 2-AG acts as ligand on CBR2 and is hydrolyzed by MAGL. The endocannabinoid system is involved in stress-associated pain, as seen in irritable bowel syndrome [101]. To address the role of the endocannabinoid system on TJPs and permeability, rats who underwent chronic water avoidance stress (1 h/10 days) were treated by intragastric injection with JZL184, a selective MAGL inhibitor; and/or with SR141716A, a CBR1 antagonist; or with AM630, a CBR2 antagonist [102]. The data evidenced that stress increased MAGL and gut permeability measured with fluorescein isothiocyanate–dextran (FD4) but decreased 2-AG protein expression. Outcomes of stress on 2-AG and on TJP expression (cldn-1, cldn-2, cldn-5m and occludin) and on intestinal permeability were reversed by JZL184, but the administration of SR141716A abrogated the effects of JZL184. The endocannabinoid 2-AG is an anti-nociceptive lipid with effects on gut permeability, but its degradation by MAGL is detrimental to the gut permeability regulation; thus, administration of selective CBR1 antagonists to block MAGL degradation upon 2-AG may represent a potential pharmacologic intervention to control the stress-induced MAGL outcome on the gut barrier breakdown in human diseases of clinical relevance [102].

(iii) Mast cells are pivotal players of stress-induced gut permeability dysfunction, as seen in irritable bowel syndrome [103]. Stress-induced mast cell activation results from a direct pathway (peripheral nervous fibers) or indirectly via the HPA and sympathetic adrenal–medullar axes. Mast cell activation takes place through the ligation of mast cell membrane receptors with bioactive molecules released by enteric and extrinsic afferent nerves; among these are CRH, catecholamines (noradrenaline and adrenaline), and neuropeptides such as vasoactive intestinal polypeptide (VIP), nerve growth factor (NGF), calcitonin gene-related peptide (CGRP), and SP major nociceptive mediator. Mast cell activation leads to degranulation and the release of a plethora of potent bioactive molecules such as mast cell tryptase, a key factor that disrupts the intestinal barrier and inflammatory cytokines like TNFα, among many others [103,104]. It is known that the stress-induced TNFα response drives TJPs dislocation, and pro-apoptotic and pro-oxidative intermediaries, leading to increased gut permeability [22,105]. In rats experiencing chronic water avoidance stress (1 h/7 days), experimental studies have shown that the colonic mast cell infiltration and permeability increased, as evidenced by the TEER decrease and HRP (44 kDa) flux increase. Furthermore, TNFα, IL-1β, and cldn-2 mRNA levels increased, whereas ZO-1 and junctional adhesion molecule-A (JAM-A) levels decreased; moreover, a significant correlation between colonic cldn-2 and IL-1β mRNA levels was seen [106]. It seems that mast cell infiltration in the colon, but not in the small intestine, is a sensitive marker of both acute and chronic stress, as documented in rats who underwent water avoidance stress [20,79].

Additional experiments support the role of mast cells in stress-induced intestinal permeability alterations, as documented in stressed mice who experienced acute restraint/acoustic stress or in mice treated with the mast cell degranulator BrX-537A and/or the mast cell stabilizer doxantrazole [107]. The effects of acute stress and BrX-537A (mast cell activator) on the increase in paracellular permeability (measured by ^51^Cr–EDTA) were either abrogated by the mast cell stabilizer doxantrazole or favored by interferon γ (*ifng*) gene, which encodes endogenous IFN-γ. Acute stress decreased ZO-2 and occludin mRNA expression, while stress and BrX-573 increased the tight junction opening and blunted the protein expression of protein kinase Cζ (PKCζ) and alkaline phosphatase as markers of colonocyte differentiation [107]. The findings indicated that mast cells disrupt the gut permeability via the IFN-γ signal pathway that drives the activation of myosin-like chain kinase (MLCK) that, in turn, leads to perijunctional actino-myosin ring contraction and TJ opening [107].

A presumable mechanism through which mast cells break down the epithelial barrier function was addressed in BALB/c mice who underwent chronic restraint stress (1 h/10 days), and this involved SP [19]. Substance P is a neurotransmitter expressed by enteric neurons and extrinsic afferent nerves involved in stress-induced paracellular permeability [18]. The mechanism entails the release of SP from nerve endings (enteric neurons and/or extrinsic afferent nerves) that activate eosinophils via neurokinin receptors (NKR1 and NKR2) to produce CRH. In turn, CRH acts via receptors on mast cells, leading to their activation and degranulation, resulting in the release of mediators that weaken the gut barrier [19]. 

Mast cell activation by stress leads to the release of mast cell tryptase (MCT), an enzyme that cleaves proteinase-activated-receptor-2 (PAR2), a transmembrane enzyme expressed on the apical and basolateral membranes of epithelial cells and afferent nerve endings [103,108]. Mast cell tryptase activates PAR2 at the basolateral side to trigger the redistribution of ZO-1 and occludin, causing the translocation of perijunctional F-actin to cytosol via β-arrestin (β-ARR), via the extracellular signal-related kinase 1/2 (ERK1/2) dependent pathway [108]. Furthermore, PAR2 activation leads to intestinal permeability and ZO-1 disruption that takes place under IFN-γ-induced inflammation or in a non-inflammatory environment. Both conditions involve the activation of calmodulin, which binds and activates MLCK; MLCK causes tight junction opening by perijunctional ring myosin phosphorylation [109] (Figure 3, mechanisms 1 and 2).

(iv) Glucagon-like peptide-2 is an enteric peptide that is co-secreted with other gut peptides (GLP-1, cholecystokinin, peptide YY, neurotensin) in response to nutrient ingestion by enteroendocrine L cells localized throughout the gastrointestinal tract length, but particularly in the distal ileum and colon [110]. GLP-2 modulates mucosal permeability, apoptosis, and cell proliferation through its ligation with the G-protein-coupled GLP-2 receptor (GLP2R) expressed in enteric neurons from the small and large intestine. Murine models of chronic water avoidance stress evidenced the effect of GLP-2 (injected subcutaneously) on colonic gut barrier [16]. Stress-induced effects on the increase in macromolecular permeability (measured with HRP 44 kDa), bacterial colonic adherence, inflammatory cell infiltration, and passive permeability, as well as ion transport (in Ussing chambers), were significantly blunted by GLP-2. Although TJP assessment was not documented in this contribution, the finding suggested the potential application of GLP-2 in therapeutic interventions to control stress-associated gut permeability disfunction. An overview of the mechanisms underlying the stress-associated pathways that regulate barrier permeability and TJP expression is depicted in Figure 3.

## 5. Clinical Implications of Epithelial Junctions and Intestinal Permeability

In clinical practice, structural and functional alterations in the gut barrier are strongly associated with the development and severity of some intestinal diseases like IBS and IBD. The clinical manifestations and severity of these diseases are conditioned by changes in the epithelial integrity (including epithelial junction and intestinal permeability), proinflammatory immune responses, reorganization of intestinal vasculature, and intestinal motility. 

Irritable bowel syndrome is a common, chronic gastrointestinal condition that is clinically characterized by disturbances in bowel habits (diarrhea or constipation) and abdominal pain, in the absence of a known organic pathology. Several underlying factors are involved in IBS, including genetic predisposition, environmental interactions such as familial susceptibility, and psychosocial stressors (depression, anxiety, or somatization). Development of IBS symptoms are related to an inflammatory response or an infectious process that may alter intestinal permeability along with the microbiota, initiating a cascade of events (e.g., infiltration of inflammatory cells such as T cells and mast cells, localized edema, and the release of cytokines or chemokines) [111,112]. In some patients with IBS, the intestinal permeability may be altered, and this process is associated with the potential alteration of the local release of serotonin that modulates sensory and motor functions [112,113]. In addition, circulating levels of proinflammatory cytokine TNFα correlate with symptoms and anxiety in IBS-D patients [114]. Mechanistically, diarrhea and pain in IBS patients may result from a significant decrease in TJPs, in zonulin-1 mRNA expression, and possibly, in zonulin signaling through PAR2 [11,115]. Evidence for the presence of enlarged spaces between epithelial cells and cytoskeletal condensation in gut biopsies of patients with IBS-D has been provided by electron microscopy [116]. Furthermore, the excessive passage of macromolecules from the luminal to the basolateral side of gut tissue in biopsies obtained from patients with IBS has been demonstrated in Ussing chamber assays, which correlate with structural epithelial barrier defects [116].

Inflammatory bowel disease is a clinical condition characterized by intestinal chronic inflammation, and it includes ulcerative colitis and Crohn´s disease. Ulcerative colitis is characterized by inflammation and ulcers along the lining of the colon and rectum, and Crohn’s disease is characterized by inflammation of the lining of the small intestine. Although the underlying mechanisms are not fully known, it is likely that epithelial barrier dysfunctions or the interactions of some pathogens like the adherent invasive bacteria *Escherichia coli* with intestinal epithelial cells promotes the overproduction of inflammatory cytokines [114]. This inflammatory response increases local intestinal injury characterized by the overexpression of NFκB with the consequent production of IL-1ꞵ, TNFα, and IFN-γ, generating a vicious cycle with newer production of proinflammatory cytokines [114]. Accordingly, proinflammatory cytokines cause a reorganization or down/up-modulation of TJPs (zonulin-1, JAM-A, occludin, cldn-1, and cldn-4) and alterations in intestinal permeability. In turn, this enables the translocation of intestinal microbiota and, concomitantly, intestinal inflammation, which precedes the exacerbation of the clinical manifestations on IBD [11,114,117]. Markers of intestinal permeability (zonulin), measured using the serological method and confocal laser, are used for diagnosis in IBD patients [11,114,117].

## 6. Perspectives

Pharmacologic drugs have been formulated to control stress-induced gut barrier dysfunction [118], including lubiprostone, a synthetic prostaglandin E1 (PGE1) derivative. Lubiprostone is a bicyclic fatty acid that activates the type 2 chloride channel (CIC-2) expressed at the apical membrane of the epithelial cells. It promotes the passage of water into the luminal space and bowel movements, which can improve symptoms associated with constipation [119]. Lubiprostone seems to reverse the effects of stress on the down-modulation of GCR binding to the occludin promotor and the down-modulation of GCR and its co-chaperone HSP90 proteins. This results in the alleviation of visceral hyperalgesia and the re-establishment of gut permeability [120]. Lubiprostone is included within the list of drugs for the treatment of stress-induced gut-barrier dysfunction found in IBS, whose clinical manifestations includes constipation, bloating, and abdominal pain [119].

A wide array of intestinal proteases contributed to an increase in the gut permeability by degrading TJPs both intra- and extracellularly or indirectly via PARs [121]. One of the most characterized proteases derived from mast cell degranulation is rat mast cell protease-II (RMCP-II), a proteolytic enzyme that degrades TJPs contributing to a stress-induced gut permeability increase [122,123,124]. A synthetic protease inhibitor, Camostat mesitilate, tested in an experimental setting in rats, has proved to inhibit fecal proteases, including serin proteases [125]. It may have a potential application where feces have a high content of proteases, as found in patients with IBS [121].

Pharmacologic molecules provide an undeniable therapeutic effect, but their use is a double-edged-sword due their toxicity; hence, the search for effective and “sustainable” interventions that have been tested in experimental models has been propelled. The potential therapeutic effects of non-pharmacologic molecules, including probiotics [91], amino acids [95], and natural antioxidants, against gut barrier disturbances under stress conditions have been tested [126,127]. However, clinical studies are necessary to demonstrate the efficacy of probiotics, honey, and herbal preparations in intestinal inflammatory diseases in humans.

Probiotics represent a sustainable alternative to alleviate stress disarrangements on gut barrier permeability [91,95]. The therapeutic effect of probiotics is related to their capacity to counteract a wide array of detrimental effects of stress on TJP expression, gut permeability, oxidative intermediaries, and proinflammatory cytokines [91,95].

Oxidative intermediaries derived from stress-associated enzyme activation display a detrimental outcome on gut permeability [99,128]; thus, cysteine may have a potential beneficial effect by displaying a potent antioxidant effect against stress-induced oxidative intermediaries that disrupt gut permeability, as documented in vitro in Caco-2 cell cultures [95].

Potential products to control stress-associated gut barrier dysfunction that have been tested in rats who underwent water avoidance stress (1 h/10 days) include an herbal preparation, namely Shuganyin decoction. The beneficial effects of this decoction on gut barrier restoration was associated with its ability to block mast cell degranulation and to down-modulate the expression of PAR2 and TNFα [126]. Honey provides benefits in terms of alleviating stress effects on the gut barrier, as documented in mice who experienced chronic unpredictable mild stress. The findings indicated that, under stress conditions, honey increased the TJ complex and hepatic total antioxidant capacity (TAC) protection associated with the inhibition via the NFκB signal of oxidative markers like malondialdehyde, nitric oxide, nuclear factor (erythroid-derived 2)-like 2 (NrF2), and TNFα [127].

Experimental studies in animals open the way to continuing to investigate the effects of new pharmacological and non-pharmacological treatments in intestinal diseases aggravated by stress. This emphasizes the need to create new therapeutic alternatives for those patients who are resistant to or have a low response to current medical treatments.

## 7. Conclusions

Certain therapeutic interventions based on pharmaceutical products and herbal-derived compounds have therapeutic effects on intestinal permeability in IBS and IBD patients where stress is a risk factor.

## Figures and Tables

**Table 1:** Markers to measure the intestinal permeability from lumen toward the blood.

Marker (MW) ^1^, Administration Route and Permeability Pathway	Sample and Method of Detection	Comments
Sugars Mannitol (182.2 g/mol), oral route, TCP ^2^ L-rhamnose (164.16 g/mol), oral route, TCP Lactulose (342.3 g/mol), oral route, PCP ^3^ Cellobiose (342.3 g/mol), oral route, PCP [52]	Urine GC ^4^ and/or HPLC ^5^	Combination of lactulose/mannitol (L/M) determinates both TCP and PCP; mannitol/L-rhamnose to evaluate TCP and lactulose/cellobiose to evaluate PCP [53]. L/M are degraded by the colonic microbiota, therefore is not recommended to evaluate the colonic permeability [54]. L/M test does not reflect damage of the TJPs ^6^ [55].
Fluorochrome FITC ^7^-dextran conjugate (3–2000 kDa), oral route, PCP [45]	Plasma Fluorescence spectroscopy	Permeability to high size of FITC-dextran reflects damage to TJPs structure [55]. The standardization to detection is difficult due depends critically on the dextran molecular weight [45].
Dye EB ^8^ (960.8 g/mol), jejunal loop injection, PCP [56]	Blood Spectrophotometry at λ = 620 nm	Extent of tissue damage can be visualized macroscopically. After administration, some tissues stain strongly stained so that are useless for histological evaluation [56].
Polymers PEG ^9^ (200–8000 Da), oral route, PCP [57]	Urine LC-MS ^7^ method	PEG probe is suitable marker for gut permeability evaluation in humans [57].

^1^ MW, molecular weight; ^2^ TCP, transcellular pathway; ^3^ PCP, paracellular pathway; ^4^ GC, gas chromatography; ^5^ HPLC, high-performance liquid chromatography; ^6^ TJPs, tight junction proteins; ^7^ FITC, fluorescein isothiocyanate; ^8^ EB, Evans blue; ^9^ PEG, polyethylene glycol.

**Table 2:** Markers used to assess intestinal permeability from the blood to the intestinal lumen.

Marker (MW) ^1^, Administration Route and Permeability Pathway	Sample and Method of Detection	Comments
HAS ^2^ (67 kDa) (endogenous marker), PCP ^3^ [45]	Stool samples Standard ELISA ^4^ test	Blood levels must be normal. Not useful in end-stage liver disease and kidney disease [45].
Alexa Fluor 488 conjugate BSA ^5^ (66 kDa), I.V. ^6^ infusion, PCP [58]	Intestinal tissue Fluorescent microplate reader	This intestinal permeability test is usually used to evaluate the effects of drugs on intestinal permeability [45].
EB ^7^ (960.8 g/mol), I.V. infusion, PCP [56,59]	Intestinal tissue Spectrophotometry at λ = 620 nm	Relatively simple and affordable assay. EA dye is altered by the concentration of BSA in blood that passes through the epithelial tissue.
Radioactive chromium bound to EDTA ^8^ (^51^Cr-EDTA) (51.9961 g/mol), I.V. infusion, PCP [24,60]	Intestinal tissue After radiation of 0.12 mSv, ^51^Cr-EDTA is measured by gamma-counting	The strong binding of ^51^Cr with EDTA prevents its interaction with other ions.

^1^ MW, molecular weight; ^2^ HSA, human serum albumin; ^3^ PCP, paracellular pathway; ^4^ ELISA, enzyme-linked immunosorbent assay; ^5^ BSA, bovine serum albumin; ^6^ I.V., intravenous, ^7^ EB, Evans blue; ^8^ EDTA, ethylenediaminetetraacetic acid.

## Data Availability

Data sharing not applicable.
